# General Approach for Introduction of Various Chemical Labels in Specific RNA Locations Based on Insertion of Amino Linkers

**DOI:** 10.3390/molecules181214455

**Published:** 2013-11-25

**Authors:** Dmitri Graifer, Galina Karpova

**Affiliations:** 1 Laboratory of Ribosome Structure and Functions, Institute of Chemical Biology and Fundamental Medicine, Siberian Branch of the Russian Academy of Sciences, Prospect Lavrentieva 8, Novosibirsk 630090, Russia; 2 Department of Molecular Biology, Novosibirsk State University, Ul. Pirogova 2, Novosibirsk 630090, Russia

**Keywords:** ribonucleic acids, amino linkers, chemical synthesis, sequence-specific modification, reporter groups

## Abstract

Introduction of reporter groups at designed RNA sites is a widely accepted approach to gain information about the molecular environment of RNAs in their complexes with other biopolymers formed during various cellular processes. A general approach to obtain RNAs bearing diverse reporter groups at designed locations is based on site-specific insertion of groups containing primary aliphatic amine functions (amino linkers) with their subsequent selective derivatization by appropriate chemicals. This article is a brief review on methods for site-specific introduction of amino linkers in different RNAs. These methods comprise: (i) incorporation of a nucleoside carrying an amino-linker or a function that can be substituted with it into oligoribonucleotides in the course of their chemical synthesis; (ii) assembly of amino linker-containing RNAs from short synthetic fragments via their ligation; (iii) synthesis of amino linker-modified RNAs using T7 RNA polymerase; (iv) insertion of amino linkers into unmodified RNAs at functional groups of a certain type such as the 5'-phosphates and N7 of guanosine residues and (v) introduction of an amino linker into long highly structured RNAs exploiting an approach based on sequence-specific modification of nucleic acids. Particular reporter groups used for derivatization of amino linker-containing RNAs together with types of RNA derivatives obtained and fields of their application are presented.

## 1. Introduction

RNAs are key biopolymers involved in life and reproduction of all organisms, and information concerning interactions of RNAs with their molecular partners (isolated biopolymers or complex supramolecular ensembles) occurring in the course of a number of essential biological processes is crucially important. Such type information is gained by application of special tools, RNA derivatives that bear chemical labels at selected locations. These labels could be cross-linkers enabling covalent attachment of the RNA to its binding partner, fluorescent probes and spin labels making possible monitoring RNA environment and distance measurements in complexes of the RNA derivatives, and other types of reporter chemical groups.

A general approach enabling insertion of various chemical probes at selected RNA locations is based on introduction of a group containing a primary aliphatic amine function (amino linker) at the desired location. Chemical properties of aliphatic amines drastically differ from those of amine groups naturally occurring in RNA, in particular, they are much stronger nucleophiles and can be easily acylated under mild conditions when the native RNA amino groups remain completely unreactive. Therefore, chemical probe can be selectively introduced at the amino linker by using bifunctional reagents containing both acylating function (e.g., *N*-oxysuccinimide ester) and a reporter group (cross-linking, fluorescent, *etc.*). Thus, the main task at the introduction of a chemical probe at definite RNA location is to insert an amino linker at the desired RNA location, which can be achieved by using various approaches. This can be performed in the course of chemical synthesis of the RNA utilizing the respective synthon, a modified monomer containing either the chemically protected amino linker that can be deprotected after completion of the RNA synthesis, or a precursor, which can be substituted with an amino linker in the synthesized oligomer [1]. Short modified RNA fragments can be used for construction of longer RNA with amino linker-modified nucleotide at the desired location applying an approach based on enzymatic or chemical ligation of RNA segments [2,3,4,5,6]. Relatively long RNAs with modified nucleotides at selected sites can be obtained via their synthesis by T7 RNA polymerase on the appropriate DNA templates with the use of amino linker-containing nucleoside triphosphates derivatives [7].

An alternative strategy is direct introduction of amino linkers into the target RNA. This can be carried out by coupling of the linkers to functional RNA groups of a certain type, e.g., terminal phosphates or N7 of guanosine residues [8,9,10]. For selective introduction of amino linkers into desired internal RNA sites, there is an original approach that is applicable with relatively long (>100 nucleotides) highly structured RNAs. This approach is based on complementary addressed (*i.e.*, sequence-specific) modification of nucleic acids with derivatives of oligodeoxyribonucleotides complementary to a sequence adjacent to the target site. The method of complementary addressed alkylation was originally proposed by N.I. Grineva as early as in 1967 [11]. The method at first has been applied for sequence-specific actions on RNAs and DNAs [12], and then it has been utilized for insertion of an amino linker and subsequently a photoactivatable cross-linker at desired positions of RNAs possessing complex specific secondary and tertiary structures [13,14,15,16,17,18].

In this article, we present a brief description of methods for introduction of amino linkers at desired RNA nucleotides and their subsequent derivatization with chemicals that can specifically react with the inserted amino linkers not affecting unmodified RNA nucleotides; methods for chemical synthesis of amino linker-containing RNAs are mentioned only fractionally, and the approach based on complementary addressed modification of RNA is discussed in more detail. Advantages and shortcomings of the methods presented in this review are discussed through the text, and examples of RNA derivatives bearing chemically reactive and fluorescent labels obtained on the basis of amino linker-modified RNAs are mentioned together with fields of their application.

## 2. Introduction of Amino Linker into RNA in the Course of Its Chemical Synthesis

**Figure 1:**
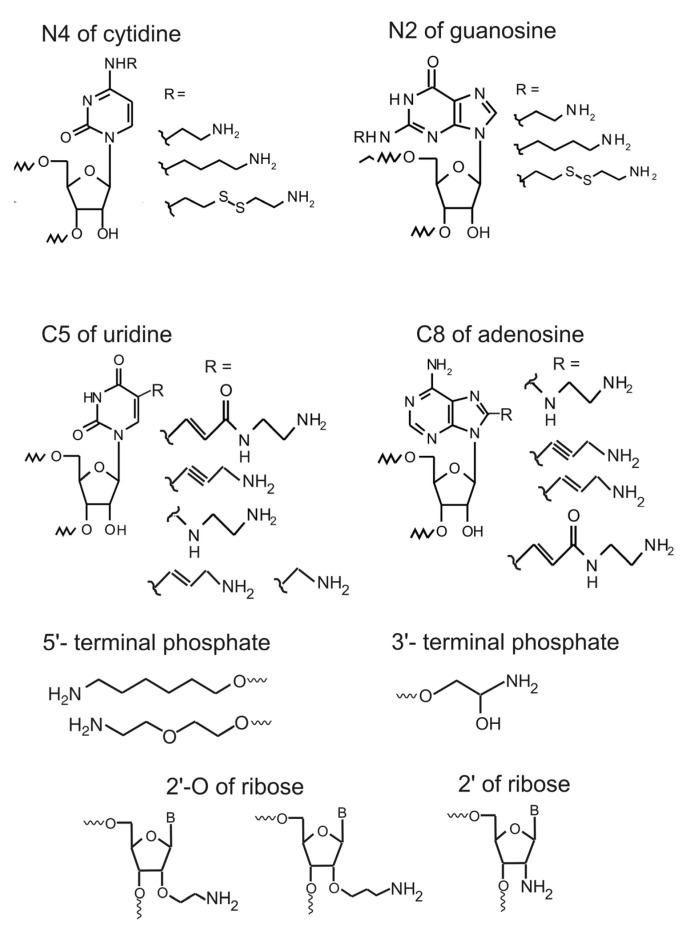
Examples of amino linker-modified RNA nucleotides grouped by the sites of the linker attachment (the respective references are given in the text).

Currently it is possible to obtain oligoribonucleotides containing amino linkers at the following positions of nucleotides: C8 of adenosine, C5 of uridine, exocyclic amino groups of cytidine (N4), adenosine (N6) and guanosine (N2), phosphate at the 3'- or the 5'-terminus, and 2'- or 2'-O of ribose [1,19,20,21,22,23,24,25,26,27,28,29,30]) (Figure 1).

Some kinds of modified oligoribonucleotides are now commercially available. Derivatives modified at the C5 of pyrimidines or C8 of purines are of special interest for studying RNA-RNA and RNA-protein interactions since these nucleotide positions are not implicated in Watson-Crick base pairing; therefore, introduction of chemical probes at these positions does not significantly impair double helical structures formed with the modified RNA and does not impede its interactions with other nucleic acids and proteins. As examples of particular approaches fruitfully used for chemical synthesis of oligoribonucleotides bearing an amino linker, we can mention those described in a series of works from Ven’yaminova’s group devoted to introduction of aliphatic diamine residues at the positions C5 of uridine, N4 of cytosine and C8 of adenosine via pre-synthetic and post-synthetic variants in the course of H-phosphonate synthesis of oligoribonucleotides. In the pre-synthetic variant, amino linker-containing synthon is incorporated into RNA chain in the course of its synthesis, in the post-synthetic variant an oligomer containing a Br- or chlorophenyl-derivatized nucleotide is synthesized with the use of the respective modified synthon, and Br- or clorophenyl group is then substituted with an aliphatic diamine [19,21,22,23]. Chemical synthesis of RNA is widely used for amino linkers introduction, but it is currently applicable only with relatively short RNAs (up to several dozen nucleotides long) [1,30]. It is worth mentioning here a recently published alternative approach for derivatization of the 5'-termini of short RNAs based on inverse electron-demand Diels-Alder conjugation, which allows selective introduction of reporter groups without amino linkers [31].

## 3. Introduction of Amino Linker into Internal RNA Positions by Assembly of the Modified RNA from Shorter RNA Segments

**Scheme 1:**
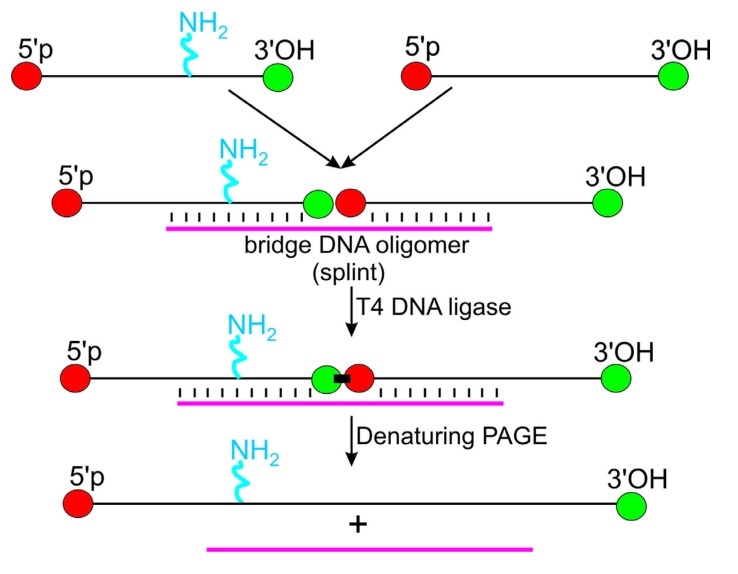
Scheme of assembly of an amino linker-containing RNA from its fragments based on splint-aided ligation. PAGE, polyacrylamide gel electrophoresis. Interactions between complementary sequences of RNA fragments and the DNA splint are indicated.

An approach to obtain RNAs containing an amino linker in a desired internal location utilizes an appropriate synthetic oligoribonucleotides bearing amino linker-modified nucleotides for assembly of the complete RNA sequences from two or more RNA fragments. This approach is based on splint-aided joining of RNA fragments by a ligase (generally, T4 DNA ligase); DNA oligomer splint serves to bring together 3'- and 5'-termini of RNA fragments to be ligated (Scheme 1). The method has been described more than two decades ago [32] and is used for RNA labeling since then. The advantage of this approach [2,3] is that it allows introduction of an amino linker at any RNA site since there are many ways to insert a linker into a short RNA fragment (see above). Instead of T4 DNA ligase, RNA ligase II or synthetic deoxyribozymes can be used for ligation of RNA fragments, although they have several shortcomings restricting their applicability [3,33]. In general, disadvantages of the discussed approach are strictest requirements to the purity of RNA fragments to be joined and low yield of ligation. The latter is probably caused by low extent of formation of correct duplexes between the RNA fragments and the DNA splint because of intramolecular structures formed by the RNA fragments; the yield can be improved by choosing optimal sites for junction [3]. Despite of the mentioned complexities, the described approach is currently widely used for assembly of modified RNAs from their short fragments, mainly to obtain derivatives for single molecule fluorescence resonance energy transfer (smFRET) experiments (for more detail, see Conclusions).

It should be noted that nonenzymatic approaches for joining of RNA fragments can be used too. An example of such approaches is Click nucleic acid ligation based on the Cu(I)-catalyzed azide-alkyne (CuAAC) reaction between RNA fragments carrying the respective modifications [4,5,6].

## 4. Synthesis of Amino Linker-Containing RNAs with the Use of T7 RNA Polymerase and Modified Ribonucleoside Triphosphates

Uridine-5'-triphosphates (rUTP) derivatized with an amino linker at the C5 or bearing 2'-amino group instead of 2'-OH retain properties of T7 RNA polymerase substrate [7,34], therefore, these derivatives can be used for the synthesis of RNA transcripts from linearized plasmid templates or chemically synthesized DNA templates. This approach is suitable for synthesis of RNA derivatives containing modified nucleotides randomly scattered over the RNA molecule. In particular, it has been used to obtain the derivatives of 5S rRNA bearing photoactivatable groups for cross-linking to 23S rRNA within the ribosome [34]. The same approach was applied to obtain synthetic model mRNAs with derivatized uridine in the AUG codon and in various positions 3'- or 5'- of it; 5-aminomethyleneuridine-5'-triphosphate was utilized for the synthesis of the amino linker-containing mRNAs [7]. The described approach has two drawbacks to be taken into account. First, modified nucleotides are incorporated into the RNA chain less effectively than unmodified ones, so to achieve desired level of incorporation, one needs to adjust optimal conditions by varying ratio of modified and normal nucleoside triphosphates used. Second, transcripts obtained with the use of T7 RNA polymerase are heterogeneous at their 3'-termini, which can lead to artifacts [3]. Besides, the mentioned approach is not suitable for site-specific introduction of reporter group into RNAs whose sequences contain several uridines. Nevertheless, amino linker-containing RNA fragments synthesized with application of the described approach can be used for assembly of longer RNA bearing amino-linkers in multiple locations in the selected RNA fragment. So, a model selenoprotein mRNA bearing aminoallyl-containing uridine residues scattered randomly over the SElenoCysteine Insertion Sequence (SECIS) element (a very specific 3'-untranslated stem-loop region of selenoprotein mRNAs responsible for incorporation of selenocysteine into the nascent polypeptide chain during protein biosynthesis) was obtained by splint-added ligation of the two RNA fragments. One of them contained the modified SECIS RNA and another contained a short 5'-untranslated region followed by the AUGUGAUUCUUC sequence encoding the tetrapeptide Met-Sec-Phe-Phe, an UAA termination codon and a part of the 3'-untranslated region [35].

## 5. Method for Introduction of Amino Linkers at the 5'-Terminus of Unmodified RNAs

**Scheme 2:**
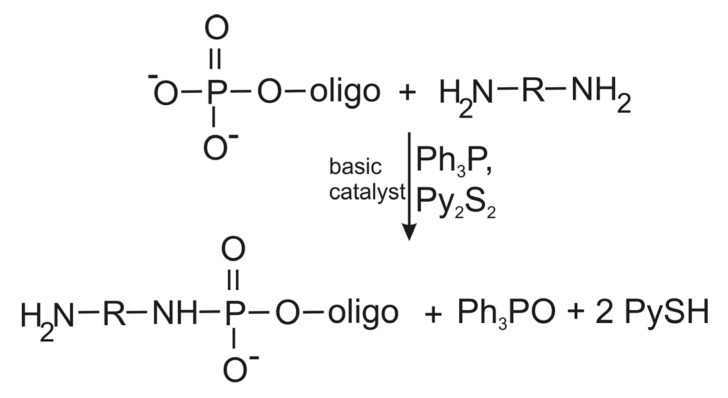
Scheme of attachment of an amino linker to the 5'-terminal RNA phosphate via phosphoramide bond by condensation with diamines exploiting a mixture of triphenylphosphine (Ph_3_P) and 2,2'-dipyridyldisulfide (Py_2_S_2_) as an oxidation-reduction condensing agent. Ph_3_PO, triphenylphosphine oxide; PySH, 2-thiopyridine.

Amino linker can be easily introduced into relatively short RNA at the 5'-terminal phosphate by its condensation with aliphatic diamines (Scheme 2). The reaction allows formation of a phosphoramide bond between the phosphate and one amine function of the diamine leaving another one free. This reaction can be performed with the use of condensing agents (typically, a mixture of triphenylphosphine with 2,2'-dipyridyldisulfide) in the presence of a basic catalyst such as *N*-methylimidazole, N,N'-dimethylaminopyridine or other in organic media (e.g., dimethylformamide or dimethylsulfoxide). RNA in this case is converted into the cetyltrimethylammonium salt prior to the synthesis to make it soluble in these solvents. This reaction was used for preparation of amino linker-containing short oligoribonucleotides utilized for preparation of photoactivatable mRNA analogs [8]. The same reaction was applied to obtain various 4-(*N*-2-chloroethyl-*N*-methylamino)benzyl-5'-phosphoramide derivatives of oligonucleotides used as mRNA analogs [36,37,38,39,40] or as tools for site-directed introduction of amino-linker into RNA [16,17,18].

## 6. Introduction of Amino Linkers at Guanosine Residues of Unmodified RNAs

**Scheme 3:**
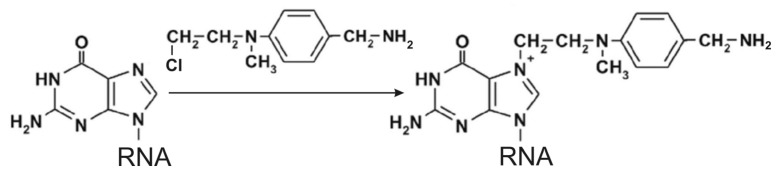
Scheme of the introduction of a 4-(*N*-ethyl-*N*-methylamino)benzylamine linker at N7 of guanosine.

An amino linker can be introduced into RNA at guanosine residues by their alkylation at the N7 with 4-(*N*-2-chloroethyl-*N*-methylamino)benzylamine (Scheme 3). This reagent in principle can also alkylate adenosines and cytidines, but reactivity of N7 of guanosines is much higher, and under appropriate conditions modification of adenosines and cytidines is almost negligible [10]. First, this method was applied to obtain photoactivatable tRNA derivatives with modified guanosines scattered randomly over the molecule [41]; distribution of the modification over tRNA guanosine residues depended on the alkylation conditions. Under conditions when the tRNA tertiary structure was unfolded, guanosines were modified in all positions, while under conditions of stability of tRNA tertiary structure guanosines participating in its folding remained untouched [9]. With application of this method, benzylamine-containing linker can be selectively introduced into RNAs whose sequences contain a single guanosine residue [10].

## 7. Approach for Site-Specific Introduction of Amino Linkers into Intact Relatively Long Structured RNAs

**Scheme 4:**
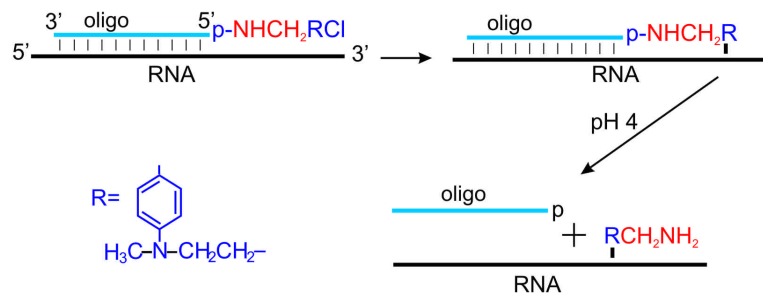
Scheme of complementary addressed (sequence-specific) incorporation of an amino linker into RNA.

An approach allowing insertion of amino linkers at practically any desirable site of relatively long natural RNAs possessing complex secondary and tertiary structures is the approach based on complementary addressed (sequence-specific) modification of nucleic acids. The first step of the approach is sequence-specific modification of RNA with a 4-(*N*-2-chloroethyl-*N*-methylamino)benzyl-5'-phosphoramide derivative of a DNA oligomer complementary to RNA sequence adjacent to the target site. Any RNA nucleotide (except for U) near the alkylating group of the deoxy-oligomer derivative bound in the heteroduplex can be modified at its nucleophilic center [12,42], and as the result, the derivative becomes covalently attached to the respective RNA nucleotide (Scheme 4). Targets of alkylation can be N7 in guanosines, C3 in cytidines and N1 in adenosines; differences in reactivities of nucleobases towards aromatic 2-chloroethylamines have no significance in the case of complementary addressed modification, and those nucleotides become modified whose positioning relatively the alkylating group of the DNA oligomer derivative bound to the RNA is the most suitable for alkylation [12,42]. Phosphoramide bond is labile in relatively mild acidic medium (pH < 4.2), and it can be hydrolysed without serious RNA damage, which results in liberation of the benzylamine group RCH_2_NH_2_ attached to the specific RNA nucleotide (Scheme 4). Applicability of complementary addressed modification for the sequence-specific introduction of amino linkers into a natural structured RNA was demonstrated at first in 80 s with tRNA^Phe^ from *Escherichia coli* as a model [13,14]. In particular, it has been shown that a pentamer derivative selectively modified the tRNA at the D stem [13] and a hexamer one at the anticodon loop [14], and that the benzylamine moieties of the derivatives attached to the tRNA could be liberated by mild acidic hydrolysis without tRNA damage [13,14].

The first studies showed that sequences that are minimally involved in formation of secondary and tertiary structure of the RNA effectively form heteroduplexes with the complementary deoxy-oligomers. The particular RNA nucleotide modified with deoxy-oligomer derivatives can be identified by reverse transcription analysis with modified RNA as a template [16,17,18] or by partial hydrolysis of end-labeled modified RNA with base-specific ribonucleases [16,43]. The first method utilizes the ability of reverse trancriptases to make stops or pauses at modified RNA nucleotides, and the second is based on inability of base-specific ribonucleases (in particular, RNAse T1) to cleave RNA chain at the modified base. Extent of the RNA derivatization depends on the extent of involvement of the target RNA sequence in formation of the secondary and tertiary RNA structures and on the stability of the heteroduplex formed by this sequence and the deoxy-oligomer. The lengths of DNA oligomer moieties chosen for the alkylating derivatives generally vary from 12 to 20 nucleotides, which allows formation of heteroduplexes stable at room temperature and makes it possible to obtain high extent of RNA conversion to the covalent adduct. With properly chosen sequences and concentrations of DNA oligomer derivative and target RNA, this extent is in the range of 30%–40%, and RNA modification occurs completely at the desired region [16,43]. However, if DNA oligomer derivative is taken in too high concentration, it can form not only perfect duplex with the target RNA sequence, but also imperfect duplexes with partially complementary RNA sequences, which can lead to undesirable modification of RNA nucleotides far away of the target site [16].

To increase the yield of RNA modification at the desired site in the cases when target sequences are significantly involved in the intra-RNA interactions, an approach was suggested that utilized helper oligodeoxyribonucleotides facilitating unfolding of RNA structure in the target sequence region. With *E. coli* 4.5S RNA as a model target RNA, it was demonstrated that two oligonucleotides complementary to the opposite strands of the RNA apical stem-loop structure stimulate synergistically the formation of a ternary complex up to 10-fold [44]. Application of this approach with -NH-CH_2_RCl derivatives of deoxy-oligomers and the same target RNA showed that helpers can increase efficiency of RNA alkylation by a factor of 6; it was also found that helper oligomer can alter specificity of target RNA alkylation. The latter effect is not obligatory: with some sequences of the target RNA, the oligomer derivative and the helper it occurs while with other does not [43]. But even in the case when helper altered the specificity of 4.5S RNA alkylation, modified RNA nucleotides were located in the expected target RNA region both with and without the helper.

**Figure 2:**
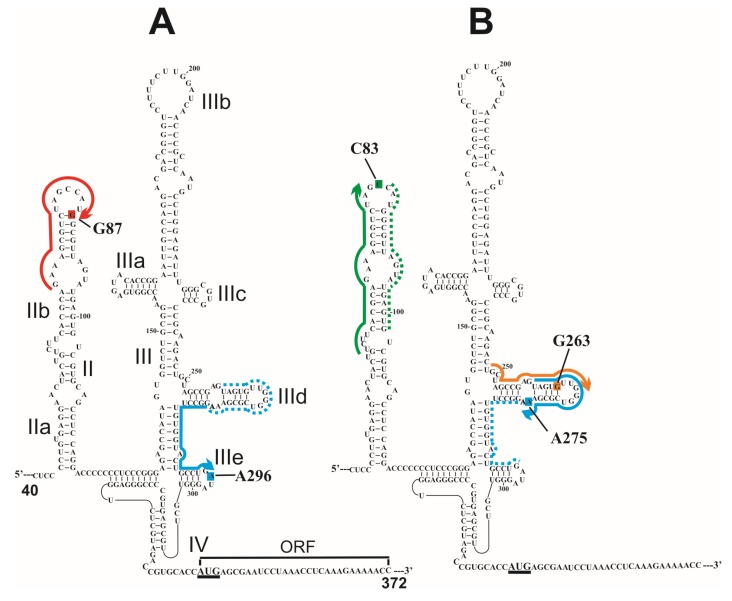
Secondary structure of the HCV IRES [45] (the initiation AUG codon is underlined, and subdomains and open reading frame (ORF) are indicated). RNA sequences complementary to deoxy-oligomers used for site-specific modification of the HCV IRES in [17] (**A**) and in [18] (**B**) are shown with thick lines with arrows indicating the 5'-phosphates derivatized with alkylating groups. RNA sequences complementary to helper oligomers used together with the alkylating derivatives are marked with dotted lines. Nucleotides of the HCV IRES modified with the oligonucleotide derivatives are shaded.

In studies with amino linker-containing IRES RNAs of the hepatitis C virus (HCV IRES) [17,18], two types helpers were used; first was complementary to the RNA strand opposite to that where the target sequence is located (as discussed above), and second was complementary to the RNA sequence adjacent to the target one. Examples of helpers used for derivatization of HCV IRES are shown in Figure 2. With one of alkylating derivatives, the target of alkylation turned out to be somewhat unexpected. One could expect that in the heteroduplex of the HCV IRES with the derivative of oligomer complementary to sequence 248–267 the alkylating group is located closer to nucleotides G265–267 rather than to G263 (Figure 2). The unusual target of alkylation in this case might be due to peculiarities of the spatial structure of the IRES and also to the low reactivity of guanosines in oligoG fragments towards aromatic 2-chloroethylamines [18] because of strong stacking interactions between the guanine residues.

In general, the method based on the complementary addressed modification of nucleic acids has no formal limitations, but technical difficulties are expected while working with RNAs > 400 nt long that can relate to low yields of RNA recovery from polyacrylamide gels upon its purification and low solubility of long RNAs in acidic medium that is necessary for hydrolysis of phosphoramide bond to occur.

## 8. Coupling of Amino Linker with Reporter Groups and Application of Derivatized RNAs

The most frequently used reaction for coupling of a reporter group to an amino linker is its condensation with *N*-oxysuccinimide esters of carboxylic acid derivatives containing the required probes. Typical derivatives for the introduction of photoactivatable cross-linker are the esters of *p*-azidotetrafluorobenzoic acid [46,47] and the ester of 4-(trifluoromethyldiazirino)benzoic acid [7,34]; the esters of dyes Cy3 and Cy5 are utilized for the insertion of the respective fluorescent dyes [48,49,50,51,52,53,54]. The condensation reaction easily proceeds at room temperature and does not touch functional groups of RNA nucleotides, providing good yields of the amino linker-containing RNA derivatization. Various oligoribonucleotides containing *p*-azidotetrafluorobenzoyl cross-linker at the desirable locations were applied as mRNA analogs to identify structural elements of the human ribosome implicated in formation of the mRNA binding center and to find peptides of translation termination factor eRF1 involved in stop codon recognition [46,47,55]. A set of HCV IRES derivatives bearing the mentioned photocross-linker at specific locations was used to determine ribosomal proteins interacting with particular RNA sites within the binary complexes of the IRES RNA with human small ribosomal subunits modeling early steps of the virus RNA translation initiation [17,18]. Finally, model selenoprotein mRNA containing *p*-azidotetrafluorobenzoylated nucleotide in its SECIS element was applied to study interactions of this element with the ribosome [35]. Diazirine-containing RNAs with derivatized uridines at specific locations were used as mRNA analogs for studying mRNA binding center of the bacterial ribosome [7], and the photoactivatable 5S rRNA derivatives were applied to investigate contacts of this rRNA with 23S rRNA within the ribosome [34]. As an example of the most widely used derivatization of amino linker-containing RNAs with fluorescent dyes, one can give the reaction of RNAs bearing 5-amino-allyl-uridines at desired locations with *N*-oxysuccinimide ester of dyes Cy3 and/or Cy5. The reaction allowed selective introduction of labels into RNA required for RNA microarray method [48], single molecule ﬂuorescence resonance energy transfer and ﬂuorescence correlation spectroscopy [49,50,52,53,54]. Besides, instead of *N*-oxysuccinimide esters, isothiocyanate derivatives can be used for coupling of reporter groups to amino-linkers attached to RNA [1,34].

As for other reactions used for coupling of reporter groups to amino linkers, the reaction with 2,4-dinitro-5-fluorophenyl azide is worth mentioning. This reaction rapidly proceeds at room temperature at pH10 without serious RNA damage. It was applied in early studies for introduction of aryl azide photo cross-linkers into tRNAs bearing amino linkers on guanosine residues [9,15,41] and the leader region of the bacterial threonyl-tRNA synthetase mRNA where amino linkers were introduced by complementary addressed modification [16]. However, application of 2,4-dinitro-5-fluorophenyl azide for derivatization of amino linker-modified RNAs was limited to the mentioned reports, probably because of restricted reactivity of this photo cross-linker, which can modify only proteins but not nucleic acids.

## 9. Conclusions

Currently relatively short RNA fragments with amino linker-modified nucleotides at desired locations became easily available due to the progress in elaboration of various approaches applicable for their chemical synthesis. Such amino linker-containing RNAs were used mainly for the subsequent derivatization with photo cross-linkers, and the resulting photoactivatable RNA derivatives turned out to be very fruitful tools for studying translational machineries of bacteria and higher eukaryotes [46,47,55,56]. Relatively long amino linker-containing RNAs obtained with the use of splint-aided ligation were successfully applied to prepare fluorescent RNA derivatives that were used in different biological tasks, e.g., elaboration of new method to measure the folding transition time of a single RNA molecule [49,50], studying conformational changes in mRNA regulatory elements known as riboswitches [52,53] related to functions of the elements, elucidation of mutual positioning of the 5'-splice site and the branch site in pre-mRNA in the course of splicing [54] and detection of tRNA levels in various human tissues [48]. Finally, application of complementary addressed insertion of amino linkers into long highly structured IRES RNA of hepatitis C virus allowed introduction photo cross-linkers at desired IRES locations, which in turn made it possible to gain crucially important information on the key role of ribosomal proteins in binding of the IRES with ribosomes at the early steps of initiation of the virus RNA translation in the infected cells [17,18]. Moreover, with the IRES RNA, a possibility of simultaneous introduction of two amino linkers at the selected RNA sites has been shown [57].

Summarizing, we can conclude that an approach based on the complementary addressed modification of nucleic acids is the most appropriate one for selective introduction of chemical reporter groups into natural RNAs containing minor nucleotides and possessing complex specific spatial structure. Since amino linker-containing RNAs obtained by this approach were used only for coupling with photo cross-linkers so far, it seems attractive to apply such RNAs for derivatization with other type reporter groups, which could be a task for further works. However, the variant of complementary addressed modification described here enables insertion of only one type of amino linker, namely, 4-(*N*-ethyl-*N*-methylamino)benzylamine, which is rather long and flexible. Although information gained with RNA derivatives obtained on the bases of the mentioned linker turned out to be in a good agreement with the related data obtained by means of other approaches [17,18], it seems that shorter and more rigid amino linkers would be preferable for works concerning studying immediate environment of particular RNA sites and distance measurements utilizing fluorescent and spin labels. Therefore, the next frontier could be elaboration of new types of reactive moieties of complementary addressed derivatives that contain the required reporter group together with a modifying function to introduce this group at a desirable RNA site directly at one step.
